# Zinc-Containing Metalloenzymes: Inhibition by Metal-Based Anticancer Agents

**DOI:** 10.3389/fchem.2020.00402

**Published:** 2020-05-19

**Authors:** Ruirong Ye, Caiping Tan, Bichun Chen, Rongtao Li, Zongwan Mao

**Affiliations:** ^1^Faculty of Life Science and Technology, Kunming University of Science and Technology, Kunming, China; ^2^MOE Key Laboratory of Bioinorganic and Synthetic Chemistry, School of Chemistry, Sun Yat-sen University, Guangzhou, China

**Keywords:** histone deacetylases, carbonic anhydrases, matrix metalloproteinases, enzyme inhibition, metallodrugs, anticancer

## Abstract

DNA is considered to be the primary target of platinum-based anticancer drugs which have gained great success in clinics, but DNA-targeted anticancer drugs cause serious side-effects and easily acquired drug resistance. This has stimulated the search for novel therapeutic targets. In the past few years, substantial research has demonstrated that zinc-containing metalloenzymes play a vital role in the occurrence and development of cancer, and they have been identified as alternative targets for metal-based anticancer agents. Metal complexes themselves have also exhibited a lot of appealing features for enzyme inhibition, such as: (i) the facile construction of 3D structures that can increase the enzyme-binding selectivity and affinity; (ii) the intriguing photophysical and photochemical properties, and redox activities of metal complexes can offer possibilities to design enzyme inhibitors with multiple modes of action. In this review, we discuss recent examples of zinc-containing metalloenzyme inhibition of metal-based anticancer agents, especially three zinc-containing metalloenzymes overexpressed in tumors, including histone deacetylases (HDACs), carbonic anhydrases (CAs), and matrix metalloproteinases (MMPs).

## Introduction

**Figure 1:**
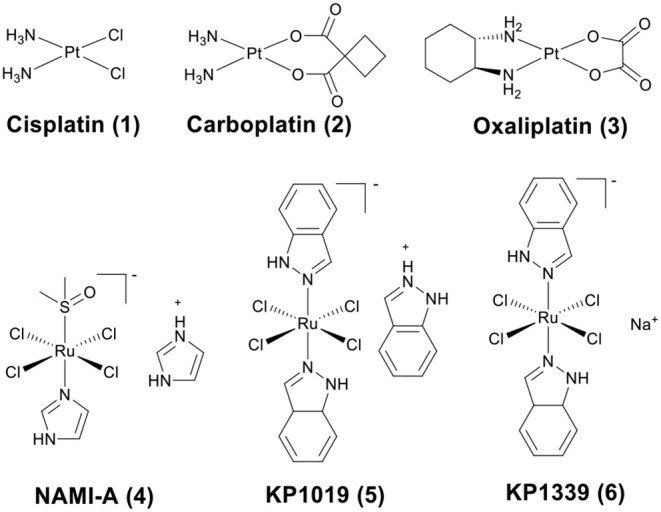
Chemical structures of platinum drugs approved by the FDA (**1**–**3**) and ruthenium complexes that have advanced to clinical trials (**4**–**6**).

The discovery of platinum-based drugs has promoted the development of metal-based anticancer agents. Platinum-based drugs, such as cisplatin (**1**), carboplatin (**2**), and oxaliplatin (**3**) (Figure 1) are being used in the treatment of ~50–70% of cancers (Bruno et al., 2017). However, the serious side-effects and easily acquired drug resistance in cancer chemotherapy of platinum-based drugs have hindered their development (Galluzzi et al., 2012). This has stimulated the exploitation of other types of novel metal chemotherapeutics, which work through different mechanisms of action and may obtain a higher therapeutic index. Numerous non-platinum-based compounds are widely studied for their potential in cancer chemotherapeutics, such as ruthenium (Zeng et al., 2017; Brabec and Kasparkova, 2018; Mede et al., 2018; Monro et al., 2019), iridium (Liu and Sadler, 2014; Caporale and Massi, 2018; Zamora et al., 2018), rhenium (Leonidova and Gasser, 2014; Lee et al., 2017; Bauer et al., 2019), gold (Mirzadeh et al., 2019; Mora et al., 2019), iron (Larik et al., 2017; Patra and Gasser, 2017), rhodium (Yang et al., 2018), and osmium (Hanif et al., 2014). Some of them have already advanced to clinical trials, such as NAMI-A (**4**) (Rademaker-Lakhai et al., 2004), KP1019 (**5**) (Hartinger et al., 2008), and KP1339 (**6**) (the sodium salt of KP1019) (Kuhn et al., 2015) (Figure 1).

DNA is considered to be the primary target of platinum-based drugs (Reedijk, 2009). However, anticancer drugs involved in the DNA binding mechanism may damage normal cells and cause serious side effects (Wilson and Lippard, 2014). Recently, research in the field of genomic and proteomics has identified various proteins or enzymes related to the survival or progression of cancer cells. Therefore, exploration of anticancer agents that target proteins or enzymes has become the preferred approach for cancer treatment (Meggers, 2009; Griffith et al., 2010; de Almeida et al., 2013; Dörr and Meggers, 2014). Many metalloenzymes have been proven to be important targets for cancer therapy, and some of these metalloenzymes contain a zinc(II) ion at the active site of the enzyme (Jacobsen et al., 2007).

**Figure 2:**
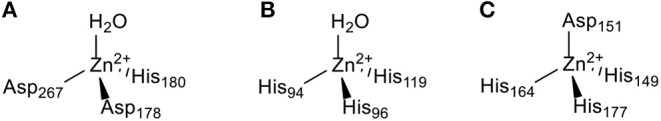
The Zn(II) ion coordination in the **(A)** HDAC8 (Finnin et al., 1999), **(B)** CA II (Eriksson et al., 1988), and **(C)** MMP2 (Morgunova et al., 2002) active site.

The zinc(II) ion plays a vital role in the catalytic and structural functions within enzymes. Numerous studies have demonstrated that zinc-containing metalloenzymes are involved in the pathophysiology and pathogenesis of various human diseases from infections to cancer. Many zinc-containing metalloenzymes, such as histone deacetylases (HDACs), carbonic anhydrases (CAs), and matrix metalloproteinases (MMPs) discussed in this review are overexpressed in human tumors. HDACs are highly expressed in lung cancer, colon cancer, prostate cancer, and breast cancer (Chen et al., 2014). CAs have been reported to be overexpressed in lung cancer, colorectal cancer, and gastrointestinal stromal tumors (Supuran and Capasso, 2015). A high level of MMPs expression has been found in cervical cancer (Yadav et al., 2014) and primary nodular melanoma (Zamolo et al., 2020). These three zinc-containing metalloenzymes are all involved in the genesis and development of cancer and have been identified as alternative targets for anticancer agents (Anzellotti and Farrell, 2008). The catalytic active centers of HDAC8 (Finnin et al., 1999), CA II (Eriksson et al., 1988), and MMP2 (Morgunova et al., 2002) are shown in Figure 2. Modulation of the activity of zinc-containing metalloenzymes with anticancer drugs has become a potential therapeutic strategy for cancer.

Metal complexes have been widely used to inhibit enzymes due to their peculiar features, such as: (i) the 3D structures of the metal complexes can fit perfectly into the hydrophobic pocket of the enzyme (Meggers, 2007, 2011); (ii) the unstable metal-ligand bond (such as halides) of metal complexes may strongly bind to amino acid side chains of the enzyme upon hydrolysis; (iii) the intriguing photophysical and photochemical properties, redox activities, and potent anticancer activity of metal complexes make it possible to design enzyme inhibitors with multiple antitumor mechanisms (Gibson, 2019). All of the above features therefore make metal complexes the ideal scaffold for enzyme inhibition.

Considering the promising antitumor potential of metal-based complexes and the importance of zinc-containing metalloenzymes, this review will focus on the recent advancements in the design of metal-based complexes as zinc-containing metalloenzymes inhibitors. Emphasis will be placed on three zinc-containing metalloenzymes of medical relevance, including HDACs, CAs, and MMPs. The design strategies of these complexes and the particular functions of the metal moiety involved will be highlighted.

## HDACs Inhibition by Metal-Based Anticancer Agents

**Figure 3:**
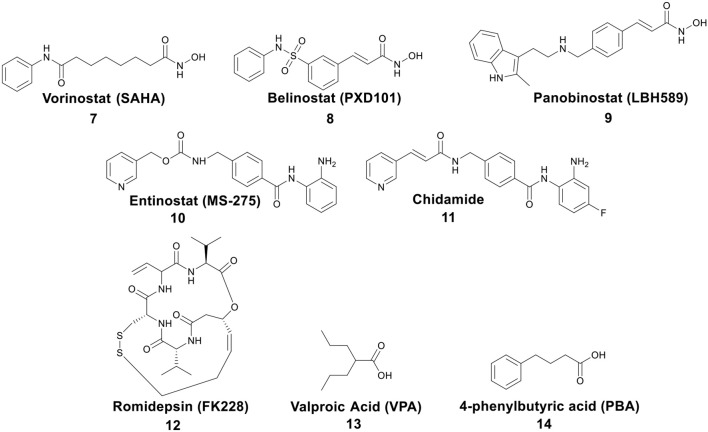
Representative structures of small molecule HDACis, among which **7**–**13** have been approved by the FDA.

**Figure 4:**
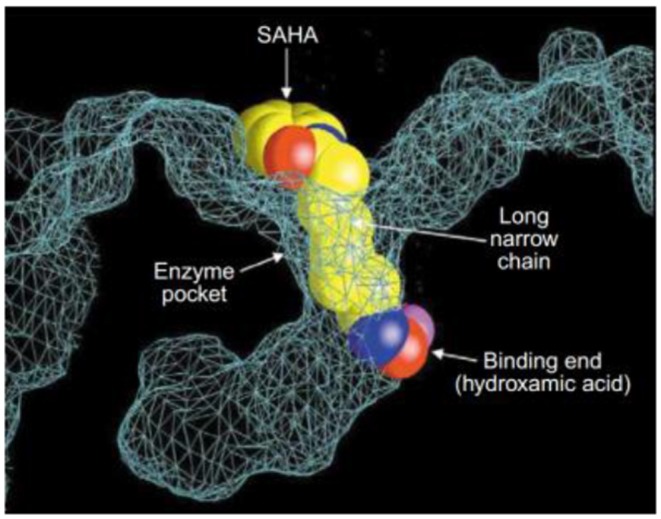
Crystal structure of a HDAC-like protein (HDLP) with SAHA (Marks et al., 2003) (Reprinted in part with permission, Copyright 2009, Elsevier).

Post-translational modifications of histones, including acetylation and methylation, are related to the epigenetic regulation of gene expression, and therefore play a pivotal role in tumorigenesis (Bolden et al., 2006). The dynamic regulation of acetylation is controlled by a pair of proteases with antagonistic functions, they are histone acetyltransferases (HATs) and HDACs. HATs cause relaxation of the chromatin structure, thereby upregulating gene transcription. In contrast, HDACs lead to chromatin condensation and transcriptional suppression (Marks et al., 2001). The overexpression of HDACs has been detected in several human tumors. Therefore, HDACs have become one of the most important targets for cancer treatment (Lin et al., 2006). HDACs inhibitors (HDACis) can promote histone acetylation and exert their anticancer activities by inducing cancer cell growth arrest, differentiation, and apoptosis (Bolden et al., 2006; Minucci and Pelicci, 2006). Several HDACis, including vorinostat (suberoylanilide hydroxamic acid, SAHA, **7**), belinostat (PXD101, **8**), panobinostat (LBH589, **9**), entinostat (MS-275, **10**), chidamide (**11**), romidepsin (FK228, **12**), and valproic acid (VPA, **13**) (Figure 3), have been approved by the FDA for the treatment of cancer, epilepsy, etc. (Marks and Breslow, 2007; Kazantsev and Thompson, 2008). SAHA is the first FDA-approved HDACi to enter the clinic, and the crystal structure of human HDAC8 complexed with SAHA (Figure 4) shows that: (i) the hydroxamic acid group (a classical Zn binding group), of general formula R-C(O)NHOH, coordinates to the active site Zn ion of HDAC8; (ii) a six carbon-long aliphatic chain occupies the enzyme's narrow channel; (iii) the phenyl-amino ketone moiety contacts at the pocket entrance, capping the pocket (Marks et al., 2003).

In the past few years, several metal-based HDACis have been developed through conjugating a known organic inhibitor with platinum, ferrocene, rhenium, ruthenium, iridium, gold, and copper moieties. These metal-HDACi conjugates inhibited HDACs efficiently and showed obvious cytotoxicity against a variety of cancer cell lines. Furthermore, most of them displayed multiple anticancer modes of action. The specific examples will be discussed in the following sections.

### Platinum–HDACi Conjugates

Numerous studies demonstrate the synergistic effect of combining HDACis and cisplatin (Stiborova et al., 2012; Diyabalanage et al., 2013). The molecular mechanisms that explain how HDACis enhance the antitumor effects of cisplatin are not fully understood. One proposed mechanism suggests that it is related to the impact of HDACis on the chromatin structure (Diyabalanage et al., 2013). HDACis may facilitate decondensation of chromatin by increasing the acetylation of histones, further promoting the formation of Pt–DNA adducts. Another possible mechanism is associated with the effect of HDACis on glutathione (GSH) synthesis (Diyabalanage et al., 2013). GSH is a major inactivator of cisplatin. It is highly expressed in a variety of cisplatin-resistant tumor cells, can combine with cisplatin to form Pt–GSH adducts, and prevent the binding of cisplatin to DNA. However, the research shows that cells pretreated with HDACis can reduce GSH synthesis quantities, thereby promoting the formation of Pt–DNA adducts.

**Figure 5:**
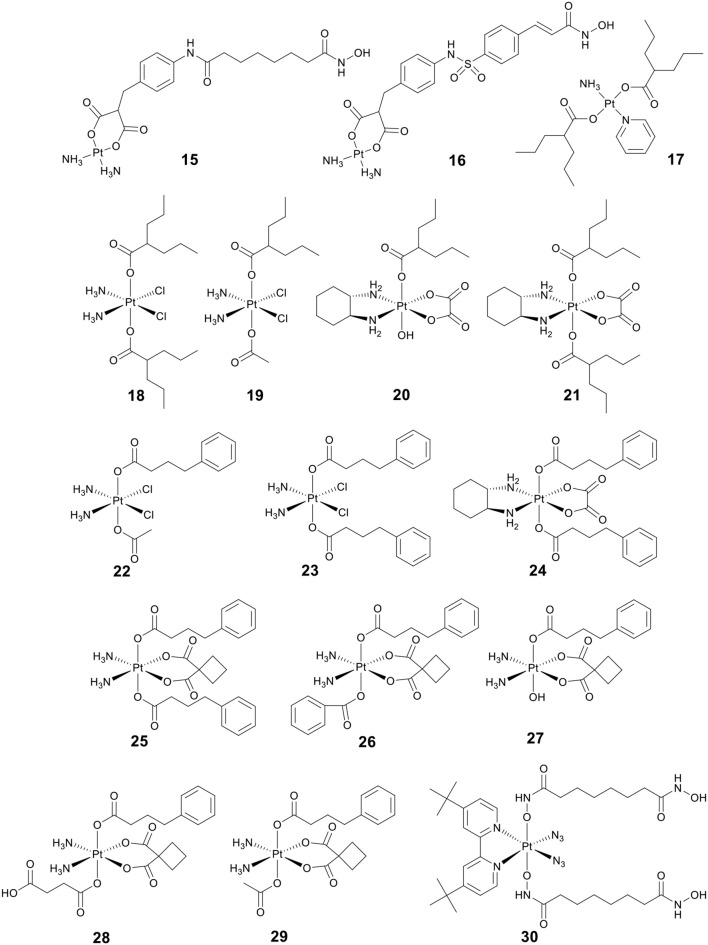
Chemical structures of platinum(II)/platinum(IV)–HDACi conjugates.

Bifunctional molecules can be prepared by incorporating an HDACi with a platinum-based DNA binding agent to enhance selectivity. Marmion and co-workers pioneered research on the development of bifunctional molecules with DNA binding and HDAC inhibitory activity (Griffith et al., 2009, 2011; Parker et al., 2013). As shown in Figure 5, the bifunctional Pt(II)–SAHA conjugate **15** was obtained by combining the malonate derivatives of SAHA (malSAHA) with *cis*-[Pt(NH_3_)_2_(H_2_O)_2_](NO_3_)_2_] (Griffith et al., 2009). The conjugate **15** displayed better selective cytotoxicity to tumor cells than normal cells as compared with cisplatin. More in-depth research indicated that conjugate **15** could accumulate in tumor cells effectively but showed weak DNA-binding ability. This was because the chlorido ligands in cisplatin were more easily dissociated than the bidentate malonato ligand of malSAHA. While, compared to in cell-free media, conjugate **15** could bind DNA more efficiently in cellulo. This was attributed to the activation of conjugate **15** in the presence of cellular GSH or thiourea (Brabec et al., 2012).

Belinostat (**8**) (Figure 3) is an analog of SAHA, which was approved by the FDA in 2014 to treat relapsed/refractory peripheral T-cell lymphoma (Poole, 2014). As a follow-up to the above research, Marmion and co-workers developed another bifunctional molecule, Pt(II)–belinostat conjugate **16** (Figure 5) (Parker et al., 2013). An *in vitro* cytotoxicity study indicated that conjugate **16** showed enhanced cytotoxicity to cisplatin-resistant A2780cisR cells compared to conjugate **15**. In addition, conjugate **16** also exhibited excellent cell selectivity compared to cisplatin and belinostat.

VPA (**13**) (Figure 3), an established antiepileptic and anticonvulsant drug (Löscher, 2002), has recently been shown to have HDAC inhibitory activity (Drummond et al., 2005). Like other HDACis, VPA can cause cell cycle arrest, cell apoptosis, metastasis, and differentiation (Duenas-Gonzalez et al., 2008). It has been reported that replacing the chlorido ligands in *trans*-platinum amine complexes with carboxylato groups can significantly increase the cytotoxicity (Benedetti et al., 2011). By combining VPA with *trans*-platinum complexes, Griffith and co-workers developed a bifunctional Pt(II)–VPA conjugate (**17**) (Figure 5) (Griffith et al., 2011). The research demonstrated that displacing the dichlorido ligands in *trans*-platinum complexes did significantly enhance the cytotoxicity against A2780 and A2780cisR.

Kinetically inert platinum(IV) complexes do not make any undesired interaction with nucleophiles before reaching tumor cells, thus avoiding the side effects associated with cisplatin and its analogs (Wexselblatt and Gibson, 2012). Pt(IV) complexes are receiving increasing attention as promising candidates in anticancer chemotherapy (Hall and Hambley, 2002; Johnstone et al., 2016). In addition, Pt(IV) prodrugs can be intracellularly reduced to active Pt(II) drugs and simultaneously release two axial ligands (Fei Chin et al., 2011). Therefore, conjugating the bioactive ligand to one or two axial positions of Pt(IV) complexes has become an extensively studied strategy for designing anticancer agents with dual or multiple mechanisms of action (Pathak et al., 2014; Petruzzella et al., 2017, 2018; Gibson, 2019).

So far, three research groups have evaluated the biological effects of Pt(IV)–VPA conjugates. In 2012, Shen and co-workers coupled VPA with Pt(IV) derivatives of cisplatin to synthesize a Pt(IV)–VPA complex, VAAP (**18**) (Figure 5) (Yang et al., 2012). VAAP showed strong synergistic cytotoxicity than the simple mixture of cisplatin with VPA against various cancer cells. VAAP was activated through intracellular reduction and released active Pt(II) and VPA, showing the similar HDAC inhibition activity with VPA. *In vivo* antitumor evaluation displayed that VAAP loaded in polyethylene glycol–polycaprolactone micelles nanoparticles could efficiently accumulate in tumors and significantly inhibit tumor growth (Yang et al., 2012).

In a similar study, Osella and co-workers also tested the cytotoxicity of VAAP against various cancer cell lines (Alessio et al., 2013). VAAP showed stronger cytotoxicity than cisplatin against pleural mesothelioma cells that are highly malignant and highly chemoresistant. This remarkable activity was attributed to the presence of the axial VPA ligands that could greatly increase the lipophilicity of VAAP, and further enhanced cellular accumulation.

By adding either one or two VPA axial ligands to the Pt(IV) derivatives of oxaliplatin, Brabec and co-workers developed another two Pt(IV)–VPA complexes, **20** and **21** (Figure 5) (Novohradsky et al., 2014). The cytotoxicity of complexes was greatly increased in cancer cell lines. Notably, **20** and **21** displayed significant cytotoxicity against both A2780 and A2780cisR cells. They exerted their antitumor activities in a dual threat manner, including DNA binding and HDAC inhibition. These results suggested that the dual targeting strategy was a viable approach in the design of platinum agents that were more effective against cisplatin-resistant cancer types.

4-phenylbutyric acid (PBA) (**14**) (Figure 3), a short-chain fatty acid type HDACi, displays potentially beneficial effects on many pathologies including cancer (Kusaczuk et al., 2015). To clarify the mechanism of action of Pt(IV)–HDACi conjugates, Gibson and co-workers prepared a series of Pt(IV) derivatives of cisplatin or oxaliplatin containing two different HDACis VPA and PBA (**18**–**24**, Figure 5), and compared their biological activities (Raveendran et al., 2016). The Pt(IV) derivatives of cisplatin with two axial PBA ligands, **23** (Figure 5), was the most potent cytotoxic agent among the compounds tested, which was 100 times more potent than cisplatin against A2780cisR. The high potency of **23** was due to the “synergistic accumulation” of Pt part and PBA. **23** showed effective HDAC inhibitory activity at levels below the IC_50_ of PBA, indicating the synergy between Pt and PBA. Mechanistically, **23** exerted multiple anticancer effects, including DNA binding, inhibition of HDACs, and caspases activation. Data also demonstrated that Pt(IV) derivatives of cisplatin containing either two axial PBA or VPA ligands were more effective than their oxaliplatin analogs.

More recently, Erxleben, Montagner and co-workers also developed a series of Pt(IV)–PBA conjugates. In their case, they chose either two PBA (**25**), or one PBA and either a benzoate (**26**), a hydroxide (**27**), a succinate (**28**), or an acetate (**29**) (Figure 5), as the axial ligands of Pt(IV) derivatives of carboplatin (Almotairy et al., 2017). Because of the higher cellular accumulation, **25**–**28** exhibited more potent cytotoxicity against all cancer cell lines screened than that of carboplatin. Complex **26** with a single PBA and benzoate as the axial ligands was the most potent complex, and it showed stronger cytotoxicity and HDAC inhibitory ability than carboplatin.

Photoactivatable Pt(IV) prodrugs can be activated upon light irradiation and produce active Pt(II) drugs, providing potential for reducing side effects (Müller et al., 2003; Min et al., 2014). Suberoyl-bishydroxamic acid (SubH) is a precursor of SAHA and also exhibits an effective HDACs inhibitory effect (Flis et al., 2009). Research has shown that SubH shows synergistic interaction with oxaliplatin in colorectal cancer cells (Flis et al., 2009). Kasparkova et al. conjugated Pt(IV)-diazido with SubH as axial ligands to synthesize a photoactivatable complex **30** (Figure 5) (Kasparkova et al., 2015). Complex **30** was inactive in the dark, but upon UV-A irradiation (365 nm), the complex was rapidly activated to release the cytotoxic Pt(II) species and HDACi SubH, and displayed more potent cytotoxicity against A2780 and A2780cisR cell lines than cisplatin. This significant activity of complex **30** was related to its inhibitory effect on HDAC, resulting in increased levels of histone acetylation. Consequently, it might make chromatin DNA more susceptible to damage caused by the platinum moiety.

### Ferrocene–HDACi Conjugates

Iron is essential for human health (Arredondo and Núñez, 2005). Iron complexes have been considered promising anticancer drugs due to their high biological activity and low toxicity. Ferrocene derivatives are an important class of iron complexes. They display diverse biological activities, including anticancer, antimalarial, antioxidant, etc., among which anticancer and antimalarial activities of ferrocene derivatives have been extensively reviewed (Larik et al., 2017; Patra and Gasser, 2017). Most drugs contain a ferrocene moiety in their structures, e.g., ferrocifen and ferroquine, which are remarkable anticancer and antimalarial agents (Patra and Gasser, 2017).

**Figure 6:**
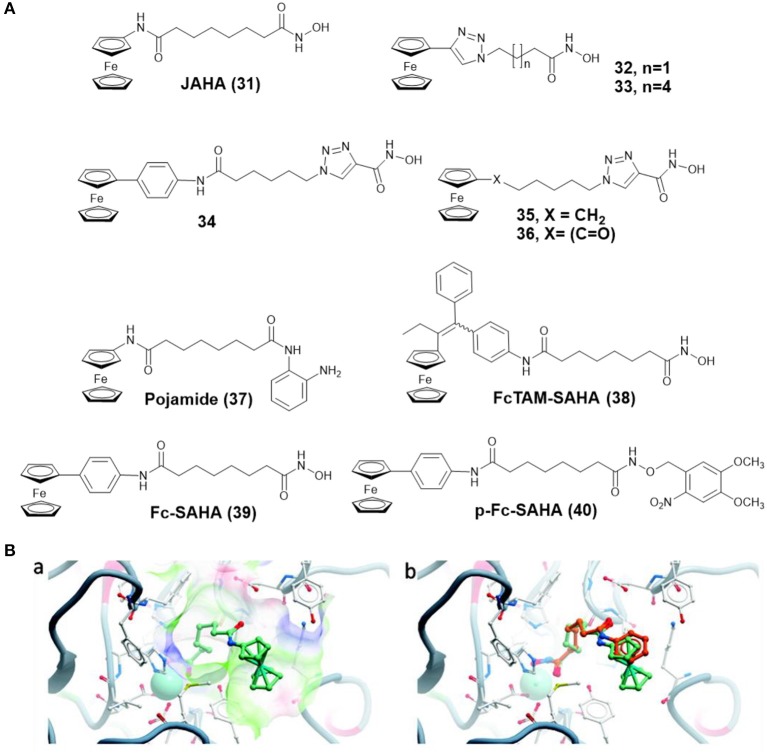
**(A)** Chemical structures of ferrocene–HDACi conjugates. **(B)** Docked JAHA and comparison to SAHA (Spencer et al., 2011) (Reproduced with permission, Copyright 2011, American Chemical Society). (a) Docked conformations for JAHA in the active site of HDAC8. (b) Comparison of JAHA to co-crystallized SAHA. The zinc ion is represented by cyan, JAHA and SAHA are shown in light green and orange, respectively.

By replacing the terminal phenyl ring of SAHA with ferrocene moiety, Spencer and co-workers prepared a ferrocene-capped HDACi, namely Jay Amin hydroxamic acid (JAHA) **31** (Figure 6A) (Spencer et al., 2011). Molecular docking studies indicated that the binding mode of JAHA in HDAC8 was similar to that of SAHA. The ferrocenyl moiety in JAHA could overlap with the aryl cap of SAHA and the hydroxamate moiety bound the catalytic zinc ion to form classical interaction (Figure 6B). Further research on HDAC inhibitory activity showed that JAHA displayed similar efficacy to SAHA.

Subsequently, Librizzi and co-workers tested the cytotoxic effects of JAHA on triple-negative MDA-MB-231 breast cancer cells (Librizzi et al., 2012). The result indicated that JAHA showed significant activity on MDA-MB-231. Treatment of MDA-MB-231 cells with JAHA led to cell cycle arrest, reactive oxygen species (ROS) generation, mitochondrial membrane potential depolarization, and autophagy inhibition. Further biological assays showed that JAHA was more selective in the expression of molecular markers related to antioxidant activity and DNA repair than the numerous changes caused by SAHA (Librizzi et al., 2017).

In another attempt, Spencer and co-workers developed a series of triazole based JAHA analogs **32**–**36** (Figure 6A) through click chemistry (Spencer et al., 2012). In this study, complex **33**, containing the triazole moiety adjacent to ferrocene cap, exhibited remarkable binding affinity with the zinc ion of HDAC8 and superior HDAC inhibition activity. Its shorter chain derivative **32** displayed a weaker inhibitory effect, highlighting the importance of chain length for HDAC inhibition. Complexes **34** and **35**, in which the triazole directly attached with hydroxamic acid group, did not show any HDAC inhibitory activity. The most effective drug, complex **33**, also inhibited deacetylation of tubulin and induced cell cycle arrest.

To further expand the chemical properties of JAHA, a ferrocene containing o-aminoanilide HDACi, namely pojamide (**37**) (Figure 6A), was synthesized by Spencer and co-workers (Ocasio et al., 2017). Pojamide displayed nanomolar potency against HDAC3 (IC_50_ = 0.09 μM). In a further examination, pojamide showed superior activity in preventing invasion of HCT116 cells. Additionally, the cytotoxicity of pojamide increased significantly after treating HCT116 cells with sodium nitroprusside/GSH. The results might be attributed to the dual mode of action of pojamide.

Ferrocifen (FcTAM) shows good efficacies against both hormone-dependent (MCF-7) and hormone-independent (MDA-MB-231) breast cancer cells (Jaouen et al., 2015). Cavaillès, Top, and co-workers developed a FcTAM–SAHA hybrid molecule **38** (Figure 6A) by combining FcTAM and SAHA (Cázares Marinero et al., 2013). Compared to FcTAM or SAHA alone, complex **38** showed a synergistic effect with an increased cytotoxicity in MDA-MB-231 cells. In MCF-7 cells, FcTAM–SAHA was more active than FcTAM, but less toxic than SAHA. Further biochemical research revealed that estrogen receptor alpha and HDAC were not the principal targets of complex **38**, which exerted its cytotoxic effects by inducing p21^waf1/cip1^ gene expression.

To reduce the side effects of HDACi, Gasser and co-workers designed a photoactivatable organometallic HDACi, p-Fc-SAHA (**40**) (Figure 6A) by photocaging ferrocene-containing HDACi (Fc-SAHA, **39**) with a photolabile protecting group (Leonidova et al., 2016). After UV-A (350 nm) irradiation, Fc-SAHA was released from p-Fc-SAHA. The HDAC assays indicated that p-Fc-SAHA showed lower activity against HDAC1, 2, and 6 than Fc-SAHA. But after light irradiation, Fc-SAHA restored the HDAC inhibition activity.

### Rhenium–HDACi Conjugates

As rhenium organometallic compounds, especially rhenium tricarbonyl complexes, offer diverse photophysical and photochemical properties that include long emission lifetimes, large Stokes shifts and resistance to photobleaching, they have mostly been used as luminescent probes (Lee et al., 2017; Otero et al., 2019). In the past few years, the anticancer potential of rhenium organometallic complexes has been widely developed and reported (Leonidova and Gasser, 2014; Ye et al., 2016; Bauer et al., 2019). Rhenium complexes can exert their anticancer activity *via* a diverse range of mechanisms, such as DNA binding (Pagoni et al., 2019), mitochondrial oxidative stress (Wang et al., 2019), phototoxicity (Leonidova et al., 2014), and enzyme inhibition (Can et al., 2012b; Wähler et al., 2014; Ye et al., 2015).

**Figure 7:**
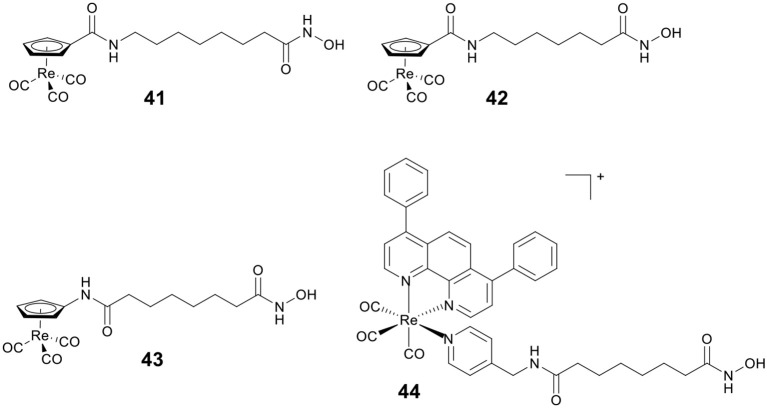
Chemical structures of rhenium–HDACi conjugates.

By replacing the terminal phenyl ring of SAHA with [(Cp)Re(CO)_3_] moiety, Alberto and co-workers designed three rhenium analogs of SAHA, **41**–**43** (Figure 7), and examined the effect of the position of amide linker on Cp ring and different linker length on the biological activity (Can et al., 2012a). Cytotoxic evaluation revealed **41**–**43** were less active than SAHA, indicating that incorporation of [(Cp)Re(CO)_3_] moiety might reduce the ability of cellular penetration. In addition, no appreciable effect on cytotoxicity was observed by changing the position of amide linker on Cp ring.

Mitochondria are vital in controlling energy production and cell death, and the dysfunctions of mitochondria are linked to tumorigenesis and tumor progression. Our group reported a Re(I)–HDACi conjugate **44** (Figure 7) with dual mitochondria targeting and HDACs inhibition (Ye et al., 2015). Mechanistic studies revealed that **44** could induce mitochondrial membrane permeabilization, ROS generation, and caspase-independent paraptosis. Notably, **44** realized its theranostic potentialities by simultaneously inducing and monitoring changes in mitochondrial morphology during paraptosis.

### Ruthenium–HDACi Conjugates

Ruthenium complexes are the most promising candidates besides platinum-based drugs (Thota et al., 2018). This is because several ruthenium complexes have entered into clinical trials, including NAMI-A (**4**) (Rademaker-Lakhai et al., 2004), KP1019 (**5**) (Hartinger et al., 2008), KP1339 (**6**) (Kuhn et al., 2015) (Figure 1), and one photodynamic therapy (PDT) agent, TLD-1433 (Thota et al., 2018). Several reviews have reported on anticancer ruthenium compounds (Brabec and Kasparkova, 2018; Liu et al., 2018; Thota et al., 2018; Rilak Simović et al., 2019). In addition, ruthenium complexes show many unique properties that make them particularly useful in drug design, for example lower cellular cytotoxicity, better water solubility, higher cellular uptake, rich physiochemical, and biological properties (Thota et al., 2018). According to their chemical structures, ruthenium complexes, currently studied as anticancer candidates, are divided in two main groups: Ru(II)-arene complexes and Ru(II)-polypyridyl complexes.

**Figure 8:**
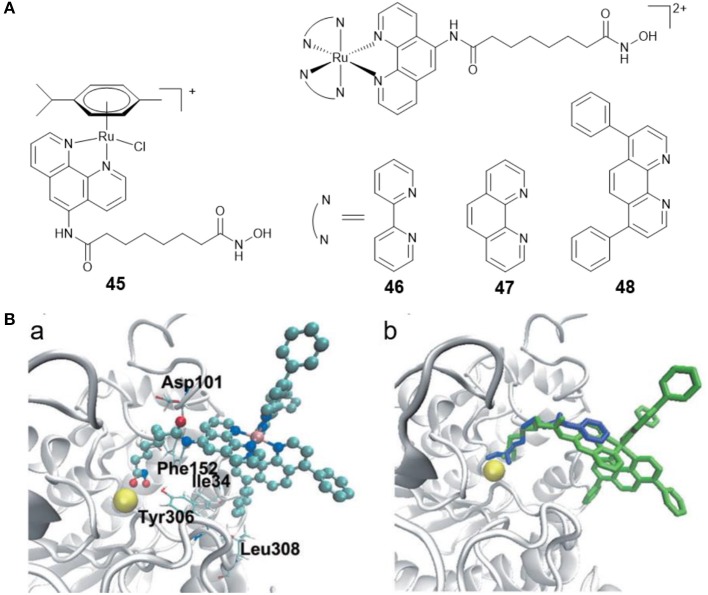
**(A)** Chemical structures of ruthenium–HDACi conjugates. **(B)** Complex **48** and SAHA docked into HDAC8 (Ye et al., 2013) (Reproduced with permission, Copyright 2013, Wiley-VCH Verlag GmbH & Co. KGaA, Weinheim). (a) Docked conformations for **48** in the active site of HDAC8. (b) Docked **48** superimposed over co-crystallized SAHA. Zinc ion, complex **48** and SAHA are colored yellow, green, and blue, respectively.

Ru(II)-arene complexes have been explored as potential anticancer agents for decades (Su et al., 2018). By coupling Ru(II)-arene moiety with a phenanthroline substituted SAHA derivative, Walton and co-workers prepared the first Ru(II)-arene HDACi **45** (Figure 8A). Complex **45** showed effective growth inhibition on the lung carcinoma cell line, and the HDAC inhibitory activity of complex **45** was comparable to SAHA (Cross et al., 2016).

Fluorescent HDACis can be used to analyze the HDAC activities, and combined with their therapeutic capabilities, can also be considered as new theranostic agents with diagnostic and therapeutic potential. According to this concept, our group reported three fluorescent Ru(II)–HDACi conjugates **46**–**48** (Figure 8A) with dual imaging and HDACs inhibition (Ye et al., 2013). *In vitro* examination showed that complex **48** was more cytotoxic than cisplatin and SAHA. Furthermore, complex **48** exhibited a strong HDAC inhibitory effect that was approximately equivalent to that of SAHA. Treating HeLa cells with complex **48** resulted in increased levels of histone acetylation. Molecular docking studies (Figure 8B) exhibited that complex **48** bound to the active site zinc ion of HDAC8 *via* the hydroxamic acid group, and Ru(II)-polypyridyl moieties were buried inside the hydrophobic pocket of HDAC8 and interacted with amino acid residues Ile34, Phe152, and Leu308. Further research revealed that complex **48** induced apoptosis in Hela cells *via* mitochondrial dysfunction and ROS production.

### Iridium–HDACi Conjugates

Iridium(III) complexes have given rise to important applications due to their rich photophysical properties (Ma et al., 2013; Ma D. et al., 2017), including their use as catalysts (Liu and Sadler, 2014) and luminescent chemosensors (Ma D.-L. et al., 2017; Kang et al., 2018). Iridium(III) complexes have been extensively studied in the search for novel metal-based anticancer drugs (Caporale and Massi, 2018; Zamora et al., 2018), as they can target different organelles (Qiu et al., 2019), and can be used as protein-protein interactions inhibitors (Leung et al., 2012) and PDT agents (Huang H. et al., 2018; Huang T. et al., 2018). In addition, iridium(III) complexes show multiple advantages over platinum and ruthenium compounds, e.g., ease of synthesis, air, and moisture stability. These features can improve their medicinal chemistry potential (Ma et al., 2014).

**Figure 9:**
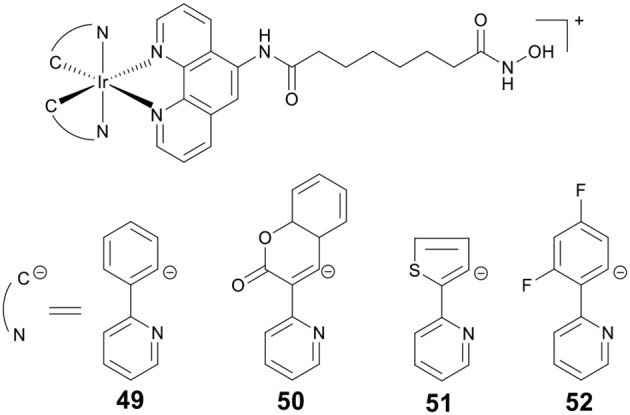
Chemical structures of iridium–HDACi conjugates.

PDT is an attractive research topic that can overcome the drawbacks of chemotherapy (Dolmans et al., 2003). More recently, our group reported four Ir(III)–HDACi conjugates **49**–**52** (Figure 9) with dual PDT and HDACs inhibition (Ye et al., 2014). Under dark condition, compounds **49**–**52** displayed moderate cytotoxicity against all cancer cells screened. However, upon UV-A (325 nm) or visible light (425 nm) irradiation, the cytotoxicity of **49**–**52** increased significantly. Compounds **49**–**52** showed potent HDACs inhibitory effects. Moreover, treatment of HeLa cells with **49** resulted in increased levels of histone acetylation, and further induced HeLa cells apoptosis *via* the inhibition of HDACs and ROS production. Upon exposure to UV-A/visible light, the anticancer activities of **49** were significantly enhanced.

### Gold Complex as HDACi

The discovery of auranofin stimulates the development of gold complexes. Recently, gold complexes have been widely studied as anticancer agents (Ott, 2009). They show strong binding affinity with sulfur, therefore thioredoxin reductase, glutathione reductase, and cysteine protease are considered as their therapeutic targets. To date, several gold complexes, such as gold complexes with N-heterocyclic carbene (Mora et al., 2019), diphosphine (Mirzadeh et al., 2019) ligands, and gold porphyrin complexes (Sun and Che, 2009), have shown effective anticancer activities.

**Figure 10:**
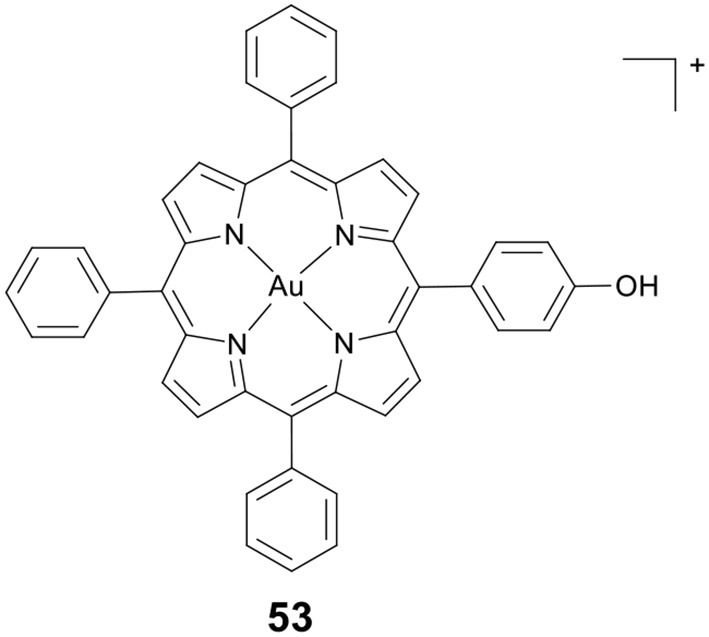
Chemical structure of Au(III) porphyrin complex.

Wang, Che and co-workers developed a novel gold(III) porphyrin HDACi **53** (Figure 10) (Chow et al., 2010). The complex displayed 100–3,000 times higher cytotoxicity than cisplatin in killing MDA-MB-231 cells. *In vivo* antitumor evaluation demonstrated that **53** significantly inhibited the growth of breast tumors. These effects were attributed to the attenuation of Wnt/β-catenin signaling by inhibiting the activity of class I HDACs. Further molecular modeling study revealed that **53** showed a different binding mode compared to TSA (a potent and specific HDACi). The side chains of **53** were not covered inside the enzyme's narrow channel. The porphyrin rings were accommodated in the shallow pocket of HDAC8 and interacted with hydrophobic residues in the pocket. The OH group of **53** pointed toward the internal cavity of HDAC8, thereby blocking the exit of cavity.

### Copper–HDACi Conjugates

Copper is an important microelement in all living organisms, it plays the role of a cofactor for many enzymes, such as cytochrome oxidase and superoxide dismutase (Bertini et al., 2010). Copper complexes are studied for many biological applications, such as anti-inflammatory, antimicrobial, antioxidative, anti-proliferative and other applications (Szymanski et al., 2012). In recent years, the antitumour potential of copper complexes has been extensively studied (Santini et al., 2014). Cu(II) is a redox active metal ion. The main biological functions of copper complexes involve redox reactions; thus, they can cause oxidative DNA damage and show excellent DNA targeting properties (Erxleben, 2018).

**Figure 11:**
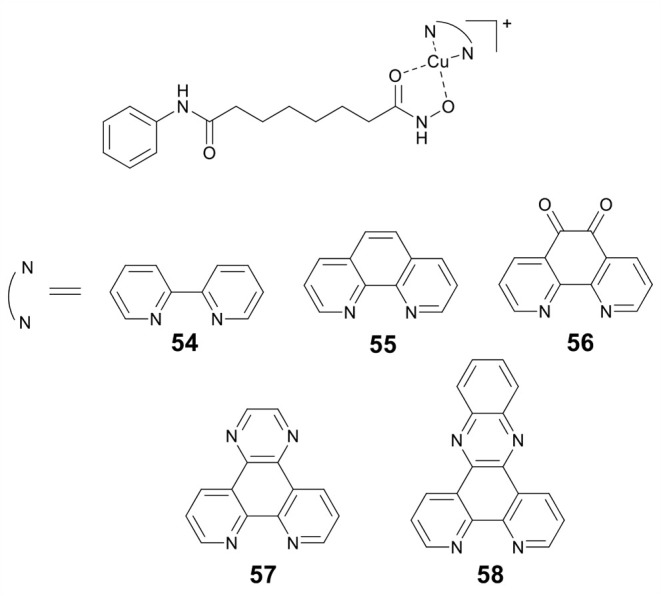
Chemical structures of copper–HDACi conjugates.

Marmion and co-workers previously reported that dual HDAC inhibition and DNA binding could be realized through conjugating SAHA to Pt(II) (Griffith et al., 2009). In this study, this group developed a series of Cu(II) prodrugs (**54**–**58**, Figure 11) containing SAHA and phenanthrene ligands as DNA intercalative components (McGivern et al., 2018). Complexes **54**–**58** demonstrated excellent DNA recognition and binding affinity and could induce DNA damage by generating ROS. As compared to SAHA alone, **54**–**58** showed enhanced cytotoxicity. Significantly, HDAC inhibition experiments revealed that two lead drug candidates **57** and **58** showed comparable inhibitory activity to SAHA after just 24 h of treatment. This revealed that the Cu(II) moiety might improve the cellular uptake of SAHA. Mechanistic studies indicated that **57** and **58** exerted their cytotoxic effects mainly *via* an apoptotic pathway.

## CAs Inhibition by Metal-Based Anticancer Agents

CAs are the most widely studied zinc-containing metalloenzymes, which catalyze the reversible hydration of CO_2_ to yield ${\text{HCO}}_{3}^{-}$ and H^+^. Many CAs isoforms play key roles in physiologic processes, including acid-base homeostasis, secretion of electrolyte, bone resorption and so on (Supuran, 2008). In the recent past, CAs have attracted attention because of hypoxia-induced overexpression of CA isozymes IX and XII in cancer cells (Hussain et al., 2007). Therefore, CA IX, and XII are regarded as the potential target for developing anticancer agents (Thiry et al., 2006). And inhibition of CAs is an area of cancer treatment that is currently being studied extensively (Supuran, 2012).

**Figure 12:**
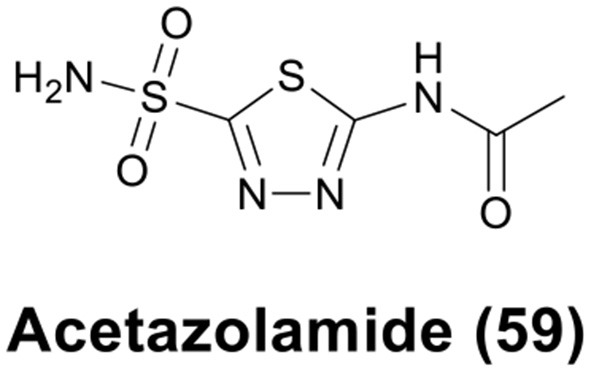
Chemical structure of acetazolamide (AAZ).

The sulfonamide group (R-SO_2_NH_2_) is one of the classical Zn binding groups of CAs inhibitors (CAis). It binds to the Zn(II) ion at the active site of CAs by deprotonation, which disrupts the normal catalytic process (Supuran and Scozzafava, 2007; Supuran, 2008). Most clinically used CAis contain the R-SO_2_NH_2_ motif, for example acetazolamide (AAZ, **59**) (Figure 12), which is the first non-mercurial diuretic used in clinical treatment (Biancalana et al., 2018).

Researchers have made many successful attempts to design more effective CAis by incorporating different moieties in sulfonamide. Metal-based compounds, e.g., ferrocene, ruthenocene, rhenium, technetium, and ruthenium, bearing the sulfonamide group have demonstrated promise as potential CAis. Some of them are 10–100 times more potent than the parent sulfonamides (Salmon et al., 2007).

### Metallocene as CAis

**Figure 13:**
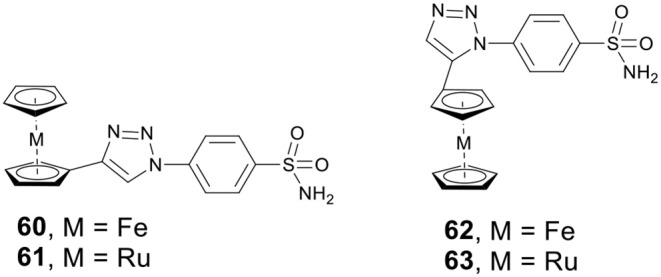
Chemical structures of ferrocene/ruthenocene CAis.

Metallocenes, including ferrocene and ruthenocene, have shown potential anticancer activity against a variety of cancer cell lines. Supuranb, Poulsen, and co-workers reported four metallocene-based CAis (**60**–**63**, Figure 13) and evaluated the impact of the metallocene tail orientation of complexes **60**–**63** on CA inhibition (Salmon et al., 2007). The result demonstrated that metallocenes with 1,4-triazole regioisomer showed higher CA IX selectivity over 1,5-triazole regioisomer. Furthermore, ruthenocenyl derivatives showed stronger CA inhibition than ferrocenyl compounds. Protein crystal structures of metallocenes with CA II showed that the sulfonamide moiety bound to catalytic zinc, and the ferrocene or ruthenocene were filled into the hydrophobic pocket of CA II (Salmon et al., 2012a). Notably, complexes **60**–**63** were more selective for cancer-related CA IX or XII than analogs containing simple benzene rings. The excellent activity and isoform selectivity of **60**–**63** might be attributed to the better matching of the metallocene's 3D structure with the hydrophobic pocket of CA II (Salmon et al., 2012b).

### Rhenium/^99m^ Technetium Complexes as CAis

**Figure 14:**
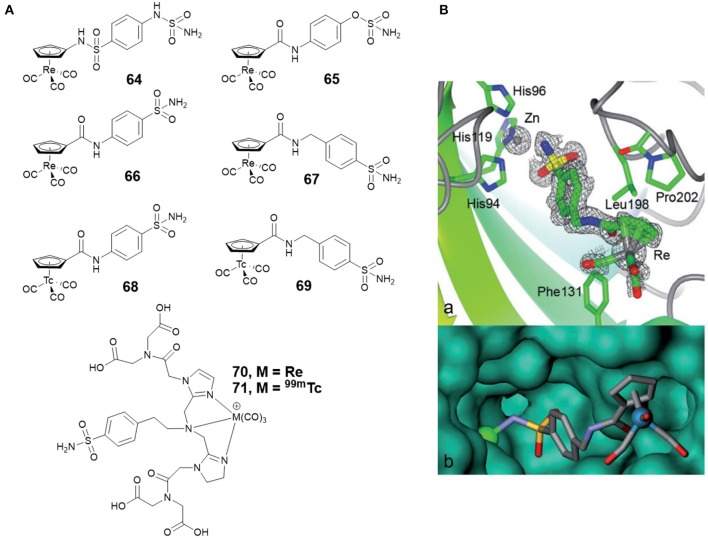
**(A)** Chemical structures of rhenium/^99m^ technetium CAis. **(B)** Co-crystal structure of **67** bound to CA II (Can et al., 2012b) (Reproduced with permission, Copyright 2012, Wiley-VCH Verlag GmbH & Co. KGaA, Weinheim). (a) Electron density map of **67** binding to the active site of CA II. (b) Detail of the binding cavity of CA II with **67**. The zinc ion appears as a green sphere.

Alberto and co-workers synthesized four new piano-stool-type Re complexes (**64**–**67**, Figure 14A) with a sulfonamide group (Can et al., 2012b). Complexes **64**–**67** showed nanomolar affinities for cancer-related CA IX and XII. The co-crystal structure of **67** with CA II displayed that the sulfonamide moiety bound to the catalytic zinc (Figure 14B). There was no interaction between the [(Cp)Re(CO)_3_] substituted tail group and the protein, but, the [(Cp)Re(CO)_3_] part interacted with the hydrophobic residues Phe131, Leu198, and Pro202 (Figure 14B). To expand the concept from therapy to theranostic, the ^99m^Tc analogs (**68** and **69**, Figure 14A) of **66** and **67** were prepared. Inert ^99m^Tc compounds exhibited identical biological behavior to rhenium, therefore, these potent CAis combinations could be applied in theranostic, in which Re is used for therapy and ^99m^Tc analogs are used for diagnosis.

Studies demonstrate that radiolabelled sulfonamides with therapeutic capabilities can not only be used to visualize hypoxic tumors, but also to inhibit the function of hypoxic tumors (Dubois et al., 2011). Babich and co-workers reported two benzenesulfonamide based CA IX inhibitors, which contained tridentate chelates complexed with the M(CO)_3_ core (M = Re for **70** or ^99m^Tc for **71**, respectively) (Figure 14A) (Lu et al., 2013). *In vitro* binding affinity showed that radiolabeled ^99m^${\text{Tc(CO)}}_{3}^{+}$ complex **71** was the most potent compound, with an IC_50_ value of 9 nM against CA IX expressing hypoxic HeLa cells. The work identified a high affinity ^99m^${\text{Tc(CO)}}_{3}^{+}$ radiolabeled CA IX inhibitor that has potential in developing theranostic agents to treat hypoxic solid tumors.

### Ruthenium Complexes as CAis

**Figure 15:**
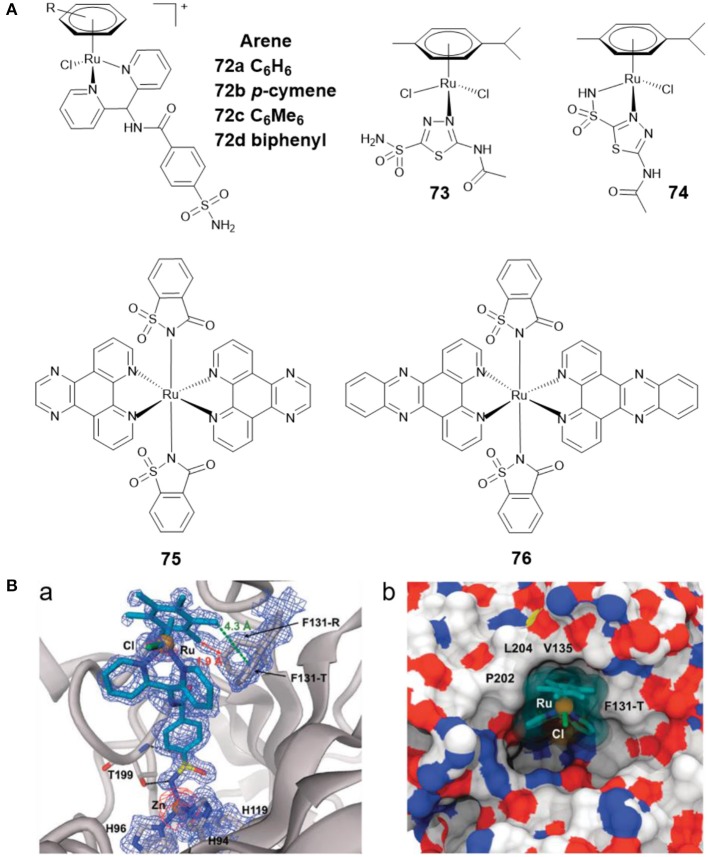
**(A)** Chemical structures of Ru(II)-arene/Ru(II)-polypyridyl CAis. **(B)** Crystal structure of **72c** bound to CA II (Monnard et al., 2011) (Reproduced with permission, Copyright 2011, The Royal Society of Chemistry). (a) Electron density map of **72c** binding to the active site of CA II. (b) Detail of the binding cavity of CA II with **72c**.

Ru(II)-arene complexes are reported to be potential catalysts and metallodrugs. Ward et al. developed four Ru(II)-arene complexes **72a**–**72d** (Figure 15A) with arylsulfonamide as CA II inhibitors (Monnard et al., 2011). The binding profiles of **72a**–**72d** toward CA II showed that **72d** exhibited the strongest affinity toward CA II. The co-crystal structure of **72c** with CA II (Figure 15B) revealed that the sulfonamide group formed the interaction with catalytic zinc, the aryl spacer interacted with the hydrophobic residues P202, L204, V135, F131-T, and Ru(II)-arene moiety lay at the entrance of the cavity.

AAZ was found to reduce tumor growth when administered alone, and to delay tumor development when given in combination with other chemotherapeutics (Teicher et al., 1993). By reacting AAZ with Ru(II)-arene scaffold, Marchetti et al. developed two Ru(II)-arene compounds **73** and **74** (Figure 15A) (Biancalana et al., 2018). Both complexes showed reasonable stability in aqueous media. The antiproliferative behavior of the two complexes indicated that they were not cytotoxic to cancer cells A2780 and A2780cisR, nor to non-tumorigenic HEK3 cells. The CA inhibition activities of **73** and **74** were not assessed in this study. Subsequently, Supuran and co-workers tested their inhibitory effects against several CAs isoforms (Seršen et al., 2019). The results exhibited that **73** and **74** showed potent inhibitory effects against hypoxic tumor overexpressing CA IX and XII.

Ruthenium complexes are effective PDT and photoactivated chemotherapy (PACT) anticancer agents (Liu et al., 2018). Compared with chemotherapy, PDT and PACT are attractive alternatives for cancer treatment due to their strong therapeutic efficacy and minimal side effects. The anticancer drugs with dual PACT and PDT modes may obtain higher efficacy in cancer treatment. Patra et al. reported two ruthenium(II) complexes of saccharin (**75** and **76**) (Figure 15A) and explored their biological interactions and photoinduced DNA damage activity (Kumar et al., 2017). Saccharin is known to be an inhibitor of CA IX. Mechanistic studies showed that photoexcitation of **75** and **76** using UV-A (365 nm) resulted in the dissociation of the saccharin ligand, and both complexes showed significant photoinduced DNA cleavage activity. Under anaerobic conditions, the photocleavage of DNA was also found to occur through a PACT mechanism.

## MMPs Inhibition by Metal-Based Anticancer Agents

**Figure 16:**
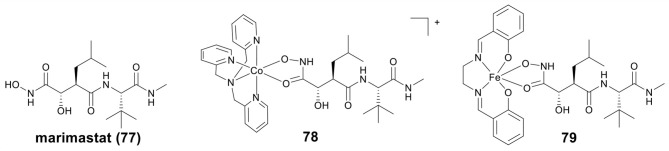
Chemical structures of marimastat and metal-containing analogs.

MMPs are important for healthy tissue remodeling and extracellular matrix protein components degradation (Jabłonska-Trypuć et al., 2016). The abnormal expression of MMPs has been found in many diseases, including tumor invasion and metastasis (Vihinen et al., 2005). Therefore, MMPs inhibitors (MMPis) have been developed to block the MMPs activity, and especially to inhibit the tumor growth and metastasis (Brown, 1995). Hydroxamic acid has also been used as a Zn binding group in MMPis (Thomas and Steward, 2000; Hu et al., 2007). Marimastat (mmst) (**77**, Figure 16) is a hydroxamate-based MMPi that has entered phase III clinical trials to treat metastatic cancer (Thomas and Steward, 2000).

Hypoxia activated prodrugs can be irreversibly reduced and release the active constituent under hypoxic conditions. Hambley et al. *first* studied two conjugates of mmst with Co(III) and Fe(III) as hypoxia activated prodrugs (Failes and Hambley, 2007; Failes et al., 2007), the structures of Co(III)–mmst (**78**) and Fe(III)–mmst (**79**) are shown in Figure 16. Take Co(III)–mmst conjugate **78** as an example (Failes et al., 2007), *in vitro* MMP inhibition assay showed that the ability of Co(III) complex **79** to inhibit MMP-9 was far lower than that of mmst alone. This was because the coordination of the hydroxamic acid group to the Co center prevented it from binding to the catalytic Zn(II) of MMP. *In vitro* cytotoxicity revealed that **78** was stable in solution before reduction and showed weak cytotoxicity against A2780 cells, but *in vivo*, **78** was activated at hypoxic tumor sites and exhibited stronger tumor growth inhibitory activity than mmst alone. Furthermore, both free mmst and **78** increased metastasis in the model used, with **78** showing even stronger inducing metastasis. Fe(III)–mmst conjugate **79** showed a higher level of MMP-9 inhibitory activity, which was related to the fact that Fe(III) complexes were more unstable than the relatively inert Co(III) (Failes and Hambley, 2007).

## Conclusions

In this review, we have classified and illustrated metal-based compounds with zinc-containing metalloenzymes inhibitory activity. As shown in these examples, most of them exhibit stronger biological activity than that of metal moiety or organic inhibitor alone, suggesting that the conjugation of known organic inhibitors to a metal center can result in a synergistic advantage. Notably, most of such complexes are significantly more active than cisplatin in both cisplatin-sensitive and -resistant cell lines, which demonstrates that inhibition of zinc-containing metalloenzymes is an effective strategy to overcome resistance to platinum-based chemotherapy. Considering the clinical success of some metal-based anticancer agents and the potential of metalloenzymes as drug targets, we expect this field to continue to flourish in the coming years.

Some major issues remain such as that most of the metal-based inhibitors reported in the literature inhibit all enzyme isoforms non-specifically (so called pan-inhibitors). It has been reported that pan-inhibitors exhibit toxicities in the clinic that may limit their potential, particularly in solid tumors (Bieliauskas and Pflum, 2008). This is mainly due to the fact that most enzymes comprise two or more isoforms, and these isoforms have generally distinct gene expression patterns and also differ in cellular localization and function. Pan-inhibitors may destroy multiple cellular internal process, which not only affects the growth and metastasis of the tumor, but also interferes with normal physiological functions, making pan-inhibitors have potentially toxic side effects. Therefore, metal-based inhibitors with strong specificity, low toxicity, and isoform selectivity may be the future of the development trend of metal-based anticancer drugs. In addition, most metal-based inhibitors reported in the literature exert their enzyme inhibitory activity by coupling with known organic inhibitors, while the metal moiety itself has difficulty working alone. Is it possible to develop simple metal complexes to exert enzyme inhibitory activity? This is also a promising research direction.

## Author Contributions

ZM, CT, and RL contributed to the design of the review. RY and BC contributed to writing the paper. All authors approved the final version of the manuscript for submission.

## Conflict of Interest

The authors declare that the research was conducted in the absence of any commercial or financial relationships that could be construed as a potential conflict of interest.
